# Social Media and Sexual Behavior Among Adolescents: Is there a link?

**DOI:** 10.2196/publichealth.7149

**Published:** 2017-05-19

**Authors:** Megan Landry, Monique Turner, Amita Vyas, Susan Wood

**Affiliations:** ^1^ Milken Institute School of Public Health Department of Prevention and Community Health The George Washington University Washington, DC United States; ^2^ Milken Institute School of Public Health Department of Health Policy The George Washington University Washington, DC United States

**Keywords:** text messaging, social media, parent-child relations, sexual behavior, adolescent

## Abstract

**Background:**

Adolescent sexual risk taking and its consequences remain a global public health concern. Empirical evidence on the impact that social media has on sexual health behaviors among youth is sparse.

**Objective:**

The study aimed to examine the relationship between social media and the change in sexual risk over time and whether parental monitoring moderates this relationship.

**Methods:**

This study comprised a sample of 555 Latino youth aged 13-19 years from Maryland, United States completing baseline and follow-up surveys. Mixed-effects linear regression was used to examine the relationship between social media and the change in sexual risk over time and whether parental monitoring moderated the relationship.

**Results:**

Sexual risk behaviors significantly increased between baseline (T1) and follow up (T2) (mean=0.432 vs mean=0.734, *P*<.001). Youth sending more than 100 text messages per day had significantly higher sexual risk scores (beta=1.008, *P*<.001) but significantly larger declines in sexual risk scores for higher levels of parental monitoring (beta=−.237, *P*=.009).

**Conclusions:**

Although adolescents exchange SMS at high rates, parental monitoring remains vital to parent-child relationships and can moderate SMS frequency and sexual risk behaviors, despite parental influence diminishing and peer pressure and social influences increasing during adolescence.

## Introduction

Adolescent sexual risk taking and its consequences remain a global public health concern. Risky sexual behaviors may lead to increased likelihood of sexually transmitted infections (STIs) and unintended pregnancies [1-3]. Adolescence, defined as 13-19 years of age, is a phase of rapid physical, emotional, and cognitive development [4]. This period is marked by an increased importance on social relationships when youth are focused on developing a sense of self and personal identity [5]. Given the importance of social relationships and the inability to fully control impulsive behaviors during adolescence [6], there may be some concern about the role social media has in adolescents’ lives.

Adolescents use mobile phones and the Web to interact with both known and unknown peers to establish and maintain social connections [7]. Such communication platforms are free of locale and time and are relatively easy to use, making these interactions a new way to foster the development of adolescents’ identity, self-expression, intimate relationships, and social well-being [8-10]. Compared with adults older than 25 years, youth between 12 and 24 years of age are the most extensive users of new technology and are more likely to be connected to the virtual world, regardless of socioeconomic status (SES), race, or ethnicity [11,12]. Social media platforms such as short message service (SMS, or texting) and social networking sites (SNS) allow self-expression, intimacy, and privacy for adolescents [10,13]. Users are able to set their own preferences to convey messages about their social identity, in the same manner that face-to-face interactions allow, but on a global scale and in contexts that are not always monitored by adults [14].

Indeed, social media is a promising channel to deliver health information, including health promotion and disease prevention messages [15,16]. However, others suggest that Internet and social media platforms might also have negative health consequences due to a false belief of privacy leading to more provocative behavior and discussion around drinking, sex, violence, suicide ideation, and bullying, coupled with less parental monitoring [9,17]. The American Academy of Pediatrics Council on Communications and Media has also argued that although social media may facilitate socialization and communication, enhance learning opportunities, and increase access to health information, it may also lead to cyber bullying or harassment, sexting, and depression [18].

Yet, there is little empirical evidence on the impact social media use has on sexual health behaviors. Landry et al [19] concluded that Latino adolescents who sent or received more than 100 SMS per day were significantly more likely to ever have vaginal sex and adolescents who logged in to a social networking account at least once per day were significantly more likely to ever have vaginal sex. Their findings are consistent with Frank [20], who reported a relationship between excessive technology use among teens and increased health risk behaviors and poorer perceived health. Teens who hyper-text (ie, send or receive more than 120 messages per day) and hyper-network (ie, 3 or more hours on social sites per day) were much more likely to be involved with unhealthy uses of technology. In fact, Frank [20] reported that 75.8% of texters and 72% of social networkers sending messages or photos that they would not want their parents to see, while 56.4% admitted to using texting or social networking to find a place to gather without parental supervision, for example, to drink alcohol (41.5%) or to meet for sex (27.4%). Additionally, Frank [20] reported that minorities, children of parents with less education, and teenagers from homes without a father were more likely to engage in hypertexting and hyper networking.

One protective factor in reducing sexual risk behaviors during adolescence is parental monitoring [21-24]. Wight, Williamson, and Henderson’s [25] longitudinal study suggested that low parental monitoring predicted early sexual activity for both sexes, and, for females, it also predicted more sexual partners and less condom use. Higher levels of parental knowledge of adolescent’s whereabouts delayed sexual onset, especially among girls [26]. Still, other investigators have linked higher parental support to a delay in sexual debut for both girls and boys [27].

Although youth are the most extensive users of new technology and are more likely to be virtually connected, regardless of SES, race, or ethnicity, there are racial and ethnic disparities in the prevalence of sexual risky behavior. For example, Latino youth often engage in riskier sexual behaviors than their White counterparts. Compared to non-Latino Whites, Latino youth have higher rates of STIs, including chlamydia, gonorrhea, and syphilis [28]. Although condom use at most recent sexual intercourse has been on the rise among adolescents (ie, from 46% in 1991 to 59% in 2013), sexually active Latino adolescents were more likely than both White and Black adolescents to not have used a condom or birth control during their last sexual intercourse [29].

Additionally, Latino youth are just as likely as their White and Black counterparts to be extensive social media users [11,30,31], so we would expect an association between social media and sexual behavior among the larger population of youth. However, the Latino youth population are unique in that they face higher sexual risk behaviors and additional risk factors such as poverty, acculturative stress, and familial and cultural barriers that potentially segregate them from the larger society and put them at even greater risk of negative behaviors [12,32,33]. Given the limited but growing body of research surrounding the impact of social media on sexual health, investigating these relationships is important for public health practice and reducing negative health outcomes.

This study used two rounds of data from a longitudinal study of Latino youth to investigate sexual risk behavior over time. We questioned whether the rate of change in sexual behavior was related to social media utilization and frequency of use over the same time. Also, it was hypothesized that parental monitoring moderates the relationship between sexual risk behavior and social media utilization, such that when parental monitoring is higher, the association between social media utilization and sexual risk behavior will be weaker, relative to when parental monitoring is lower.

## Methods

### Participants

The data for this study were derived from a sample of self-identifying Latino adolescents aged 13-19 years (mean=15.73, SD=1.03). Participants were recruited from 12 public high schools in Maryland, United States. Participants completed baseline (T1) and 16-month follow-up (T2) surveys conducted as part of a program evaluation of the *Empowering Latino Youth Project* (ELYP) between spring 2012 and fall 2013. ELYP is a 5-year cluster-randomized controlled trial of a teen pregnancy prevention program. All participants provided parental consent and youth assent to participate in ELYP. Due to the data being from an intervention study, final analyses statistically controlled for participation in the intervention or control group. The analytic sample for this study is a subsample of the entire study sample (555/873) enrolled between spring 2012 and fall 2013. Participants who completed the baseline but no follow-up survey were 25.7% (224/873) and those completing only the follow-up survey were 10.8% (94/873). Participants lost to follow up were more likely to be male, slightly older, born outside of the United States, and completed the baseline survey in Spanish. Participants lost to follow up also had statistically significantly higher sexual risk scores at baseline. Excluding participants lost to follow up from the analytic sample is underestimating the sexual risk score over time, therefore, providing a more conservative estimate.

### Data Collection

To ensure privacy and reduce reporting bias, surveys were administered via individual laptops with audio capability for youth with low-literacy levels. Study participants chose to complete the survey in English or Spanish and were given US $10 gift cards for completing the baseline survey and US $20 gift cards for the 16-month survey. The surveys were translated and back translated by native Spanish speakers affiliated with the partner community organization then pretested for readability and accuracy. Upon survey completion, the data were stored in an encrypted file to be read only by the survey design software, SNAP surveys.

### Measures

#### Social Media Use

Social media use includes SMS, Internet, and social media questions adapted from Pew Internet Project’s Teen Survey [34]. Participants were asked if they have a mobile phone, if they use SMS, and the frequency of SMS per day (high SMS: >100 per day; low SMS: ≤100 per day). Participants with a mobile phone reported the following behaviors using their phone: send or receive email, take pictures, play music, send or receive instant messages, record videos, play games, or access Internet. SMS frequency was dichotomized at 100 based on Pew data that suggest the median number of SMS per day for Hispanic adolescents is 100 [7,34]. Additionally, results from one-way ANOVA suggested the two highest texting categories (ie, 101-200 per day and more than 200 per day) had significantly different sexual risk scores compared with four lower categories (*F*_5_= 10.36, *P*<.001). Participants reported how often they exchange SMS with friends, parents, and a boyfriend or girlfriend (1 = less often or never, 2 = a few times a week, 3=at least once a day, 4=several times a day), which was dichotomized into at least once per day versus less often.

Participants were asked if they use the Internet, for what purposes, and how often (0=never, 6=several times a day). Finally, participants were asked if they had accounts on any of the following SNS and a count variable was created, such as Facebook, MySpace, Twitter, Yahoo, YouTube, Instagram, Tumblr, Google buzz, Flickr, Ustream, and other. Those with any account were asked about their frequency of logging in, which was dichotomized into daily login versus less frequent.

#### Parental Monitoring

The parental monitoring scale was adapted from Silverberg and Small [35] and validated in a Positive Youth Development Survey for Latinos [36]. The scale consisted of five items measuring participants’ perceptions of their parent/guardian’s knowledge of their whereabouts, making decisions that affect them, and seeking help or encouragement from a parent/guardian. The scale was measured on a 4-point Likert-type scale (1=never, 2=some of the time, 3=most of the time, 4=all of the time). Internal consistency was high at both survey time points (Cronbach alpha = .85 at baseline and .86 at follow-up).

### Sexual Risk Behavior

The sexual risk behavior measure used in this study was constructed based on the Centers for Disease Control’s (CDC) description of sexual risk taking [29]. The sexual behavior questions were adapted from validated measures from the US Department of Health and Human Services, Office of Adolescent Health [37] and the CDC Youth Risk Behavior Surveillance Survey [29]. The six measured variables were as follows: ever had vaginal sex (0=no; 1=yes), condom use in the last 3 months (0=used condom; 1=did not use condom), contraception use in the last 3 months (0=used contraception; 1=did not use contraception), 2 or more sexual partners in the last 3 months (0=less than 2 partners; 1=2 or more partners), individual alcohol use with sex in the last 3 months (0=no individual alcohol use; 1=any individual alcohol use), and partner alcohol use with sex in the last 3 months (0=no partner alcohol use; 1=any partner alcohol use). The individual sexual behaviors were summed to create a composite score of sexual risk behavior (baseline mean=0.425, SD=0.044, range 0-6), where higher scores indicated increased risk behavior.

### Statistical Analyses

Preliminary analyses examined frequencies and distributions of the variables of interest for the analytic sample (n=555). We first examined bivariate relationships between sexual risk behavior and mobile phone and social media use. In bivariate analyses (data not shown), there was no relationship between sexual risk behavior and access to a mobile phone, SMS with friends, or logging in to SNS once per day; so these variables were dropped from multivariate models.

Mixed-effects linear regression was used to examine the relationship between social media variables and the change in sexual risk between baseline and follow-up, while adjusting for time-varying and time-invariant covariates and allowing random effects for within and between subjects. Additionally, we examined if the relationship between social media and sexual risk was moderated at different levels of parental monitoring by entering an interaction term into the multivariate model. Final models were adjusted for gender, age, survey language, and intervention group. All analyses were conducted in STATA 12.0 (StataCorp). This study was reviewed and approved by the George Washington University Internal Review Board (IRB# 011217).

## Results

### Main Findings

Table 1 lists self-reported baseline demographic characteristics of the study sample. Participants were on average 15 years of age (mean=15.73, SD=1.03) and there were slightly more female participants (58.6%) than male participants (41.4%). The majority of respondents were in ninth grade at baseline (72.8%) and completed the survey in English (70.8%), while less than half were born in the United States (48%). On a scale from 1 to 4, participants reported above average levels of parental monitoring (mean=3.06, SD =0.748, range 1-4).

Sexual risk behaviors significantly increased between baseline (T1) and follow up (T2) (mean=0.432 vs mean =0.734, *P*<.001; Table 2). In terms of social media utilization, nearly 90% of participants used a mobile phone, SMS, Internet, and SNS at both T1 and T2. The mean number of activities on a mobile phone significantly increased between surveys (mean=5.90 vs mean=6.30, *P*<.001), while high-frequency SMS (ie, >100 per day) significantly decreased (34.0% vs 27.9%, *P*=.02). In terms of SNS accounts, Facebook use decreased over time (82.9% vs 73.0%, *P*<.001) and YouTube and Instagram use significantly increased.

**Table 1:** Study sample characteristics at baseline.

Characteristics		(N=555) n (%)	Mean (SD)
Age			15.73 (1.0)
**Gender**			
	Male	230 (41.4)	
	Female	325 (58.6)	
**Grade**			
	9th	404 (72.8)	
	10th	151 (27.2)	
**Survey language**			
	English	393 (70.8)	
	Spanish	162 (29.2)	
**Length of time in the United States**			
	US born (0)	268 (48.3)	
	0-3 years (1)	136 (24.5)	
	4-10 years (2)	102 (18.4)	
	10+ years (3)	27 (4.9)	
	Missing	22 (3.9)	
Parental Monitoring (alpha =.85) range 1-4			3.058 (0.7)

### Mixed-Effect Models for Sexual Risk From Baseline to Follow-Up

The results of mixed-effects regression analyses are presented in Table 3. The unconditional means model was estimated to calculate intraclass correlation (ICC). Results showed a statistically significant sexual risk behavior score (γ_00_=.584, *P*<.001), and participants’ mean sexual risk scores (ie, the average score across both assessments) significantly varied around the mean sexual risk score (between subjects τ_00_=.771, *P*<.001), as well as significant differences between each participants’ observed and predicted scores over time (σ^2^=.737, *P*<.001). Further, ICC calculations suggest that 49.4% of sexual risk behavior scores varied across students. This ICC value is consistent with research that suggests ICC values exceeding .40 are common in longitudinal social science studies [38]. The crude estimated sexual risk score at baseline (γ_00_=.432, *P*<.001) and the change in sexual risk score over the 16-month time period (γ_10_=.303, *P*<.001) are presented in Model 1 of Table 3. In the first multivariate model (Model 2), only variables that were significantly associated with sexual risk in bivariate analyses were included. Results from Model 2 indicate that high SMS (beta=.384, *P*<.001) and SMS to a boyfriend or girlfriend once per day (beta=.160, *P*=.01) were associated with increased sexual risk scores, controlling for age, gender, survey language, and intervention group. Higher levels of parental monitoring were significantly associated with lower reported sexual risk behavior over time (beta=−.140, *P*=.01).

**Figure 1:**
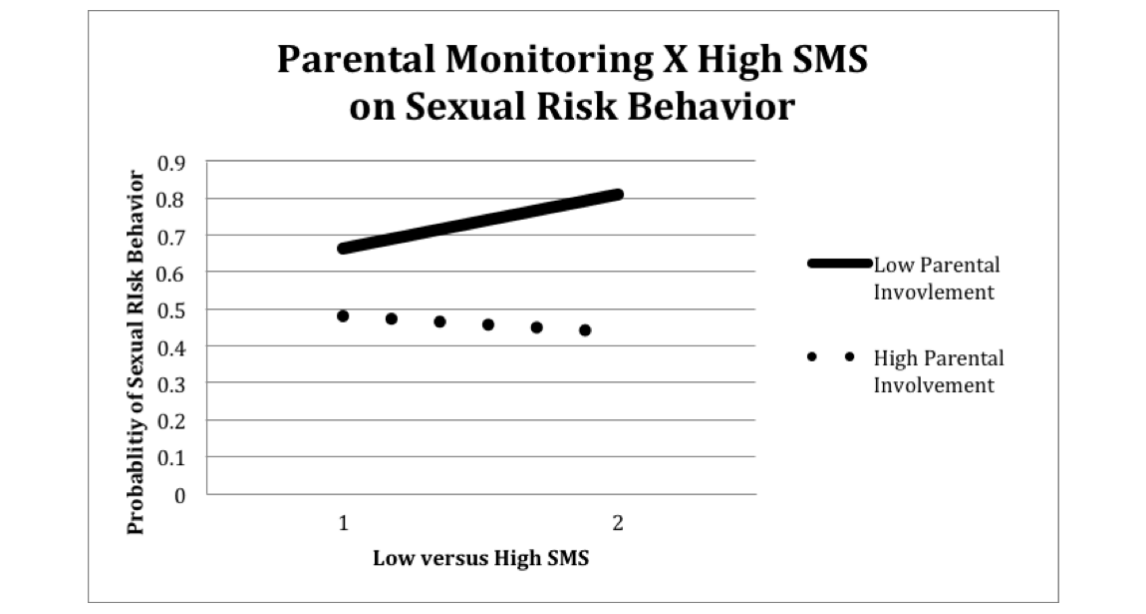
Interaction of parental monitoring and high SMS (ie, >100/day) on sexual risk behavior.

In the second set of multivariate analyses (Model 3), we extended Model 2 to include an interaction term for high SMS and parental monitoring, along with significant predictors from Model 2. Results from Model 3 indicated that, on average, sexual risk behaviors increased over time and were significantly higher for males and older youth. Further, parental monitoring interacted with high SMS. The negative interaction was graphed (Figure 1) and indicated that higher levels of parental monitoring were related to a weaker association between high SMS and sexual risk. Youth that sent more than 100 SMS per day had significantly higher sexual risk scores (beta=1.008, *P*<.001), but also experienced significantly larger declines in sexual risk scores for higher levels of parental monitoring (beta=−.237, *P*=.009).

**Table 2:** Unadjusted changes in sexual risk, social media utilization, and parental monitoring at baseline (T1) and 16-month follow up (T2).

Variables of interest and response categories		T1 % (n)	T2 % (n)	Δ T1, T2
Sexual risk composite score mean (SD) (range 0-6)		0.425 (0.97)	0.733 (1.13)	0.31 (*P*<.001)
**Social media use and behavior**				
	Mobile phone access (yes)	88.7 (488)	92.0 (494)	
Mean number of activities on mobile phone (SD)		5.90 (1.61)	6.30 (1.26)	0.4 (*P*<.001)
**SMS use**		95.7 (467)	98.6 (486)	2.88 (*P*=.007)
	More than 100 SMS per day	34.2 (149)	27.9 (135)	−6.13 (*P*=.02)
	SMS Parents at least once per day	53.4 (239)	54.1 (260)	
	SMS Friends at least once per day	87.6 (403)	86.0 (418)	
	SMS boy/girlfriend at least once per day	64.8 (283)	68.7 (322)	
**Internet use**		96.9 (533)	98.5 (529)	
	Using Internet once per day	78.2 (415)	78.6 (416)	
**Social networking account**		95.7 (528)	97.0 (518)	
	Facebook	82.9 (458)	73.0 (390)	−9.94 (*P*<.001)
	Twitter	58.7 (324)	55.8 (298)	
	YouTube	61.8 (341)	66.9 (357)	
	Instagram	14.8 (82)	27.4 (119)	12.65 (*P*<.001)
**Mean number of activities on SNS (SD)**		5.08 (0.08)	5.02 (0.09)	
	Logging in to SNS one or more times per day	79.1 (417)	82.1 (426)	
Mean parental monitoring score (SD)		3.05 (0.75)	3.00 (0.75)	

**Table 3:** Parameter estimates from mixed-effects models for change in sexual risk from T1 to T2.

Variables		Unconditional model Beta (SE)	Model 1: level 1 Beta (SE)	Model 2^a^: main effects model Adjusted beta (SE)	Model 3^a^: interaction model Adjusted beta (SE)
**Fixed effect**					
	Intercept (γ00)	0.584 (0.040) (*P*<.001)	0.432 (0.045) (*P*<.001)	0.801 (0.263) (*P*=.004)	0.758 (0.256) (*P*=.003)
	Time (γ10)		0.303 (0.046) (*P*<.001)	0.256 (0.061) (*P*<.001)	0.252 (0.058) (*P*<.001)
More than 100 SMS per day				0.384 (0.080) (*P*<.001)	1.088 (0.300) (*P*<.001)
**SMS to whom**					
	Parents			−0.084 (0.074)	−
	Boyfriend or girlfriend			0.160 (0.080) (*P*=.01)	0.148 (0.079)
Number of SNS accounts				0.018 (0.024)	−
Number of SNS activities performed				0.028 (0.024)	−
Parental monitoring				−0.140 (0.053) (*P*=.006)	−0.077 (0.064)
100 SMS × parental monitoring					−0.237 (0.098) (*P*=.009)
Female				−0.219 (0.090) (*P*=.01)	−0.218 (0.089) (*P*=.008)
Age				0.177 (0.046) (*P*=.001)	0.187 (0.046) (*P*<.001)
**Random effect**					
	Participant	0.771 (0.035)	0.785 (0.034)	0.729 (0.044)	0.746 (0.041)
	Residual σ^2^	0.737 (0.022)	0.705 (0.021)	0.616 (0.034)	0.612 (0.032)
LL ratio		−1547.279	−1523.1006	−813.11182	−853.34291

^a^ Models 2 and 3 control for age, gender, survey language, and intervention type. Age and gender were statistically significant so these variables are presented in the tables. The other control variables were not statistically significant. SMS use and having an SNS account were omitted from the final model due to collinearity with high SMS and number of SNS accounts.

## Discussion

### Principal Findings

Understanding predictors of sexual risk behavior is imperative for health and economic well-being over the life span, especially for underserved populations such as the Latino community. A plethora of studies have focused on sexual risk-taking behaviors, but with the proliferation of mobile technology and connectedness over the past decade, it is becoming clearer that social media utilization is also part of this relationship. Yet, there are still gaps in the literature with respect to social media use and sexual risk behaviors among adolescents in general. To our knowledge, this is the first study to longitudinally examine social media and sexual risk and the moderating effects of parental monitoring.

This study found a statistically significant positive association between high-frequency SMS and increased sexual risk behaviors over a 16-month period. Social media provides a context in which adolescents, who have a need for social acceptance and gratification and are still developing self-regulation skills, may find themselves vulnerable to pressures or unanticipated risk opportunities. Social media has the potential to expand and amplify existing peer relationships, which are well documented as influencing risk behaviors [39,40]. Social media may also provide increased access to partners that are more experienced, leading to increased communication about sex because of the perceived privacy of social media [41]. Thus, those who are more active on social media could partake in more risky behaviors because of a larger peer network influencing their attitudes and social norms. Although these findings indicate a decrease in high frequency SMS and Facebook use between baseline and follow up, this does not necessarily imply a reduction in overall use. Adolescents are turning to newly developed software applications (eg, apps) that allow for communication within the app. We observe this as a result of the sharp increase in a newer app such as Instagram. Other research suggests similar results of a decline in Facebook use among US youth [42].

Although this study found a statistically significant association between increased sexual risk behaviors and high frequency SMS use over a 16-month period, parental monitoring was suggested to be a protective factor in this study. Results suggest increased sexual risk among higher SMS users, but higher levels of parental monitoring moderated this relationship in the hypothesized direction. Thus, parental monitoring was associated with lower levels of reported sexual risk behaviors despite high frequency SMS. Previous studies have documented that increased communication between parents and adolescents and the greater the parent knowledge of the adolescents’ whereabouts (eg, parental monitoring), the lower the likelihood that adolescents will engage in health risk behaviors [43-46]. Specifically within the Latino community, Dittus and Jaccard [27] discovered that parental monitoring influenced delays in sexual intercourse, while Huebner and Howell [23] found parental monitoring and parenting style impacted having only one sexual partner and to use a condom. The significant finding on the impact of parental monitoring on the relationship between high SMS use and sexual risk behavior compels parents to stay involved and maintain relationships with adolescents, despite adolescence being a period of time when parental influence is diminishing and peers/social influences are increasing [47].

While social media platforms are traditionally less monitored by adults, other research has suggested that mobile phones are one mechanism for parents to maintain a relationship with their adolescents while still affording them the autonomy and self-discovery they seek during this time [48]. It is important to highlight that the parental monitoring construct measured in this study was not specific to online monitoring; rather, it assessed adolescents’ perceptions about whether their parents generally know their whereabouts and if they can go to their parents for support. This is important because in the aforementioned study, greater frequency of parental calls was associated with less adolescent-reported truthfulness, and parents calling when upset was associated with less parental knowledge and poorer family relations. Similarly, another study reported that parental social and technology supervision increased risky online activities [49]. Thus, it is important for parents to be involved in their adolescents’ lives rather than solicit information via social media platforms solely to gain control of their lives.

The findings in this study are not intended to negate the substantial benefits of using social media in public health programs. However, our findings compel practitioners, parents and youth to be practical about the risks of high frequency SMS and other connections to expansive networks, and devise strategies for harnessing social media for good. For example, social media provides an excellent platform to strengthen supportive bonds and reach underserved youth to deliver health-related content [31], but public health professionals, policymakers, and parents should also embrace programs such as those that have encouraged adolescents to remove sexual content from their social networking profiles [50]. Other potential ways for parents to get involved is using for themselves or suggesting to their children, innovative technology, such as apps around sexual health and health-seeking behaviors that are being developed at a rapid pace. For example, Hablemos is a technology-based program attempting to close the communication gap between Latino parents and children through a parent-centered tool that is culturally appropriate and aims to empower Latino parents to have discussions about sexuality and contraception [51]. Specifically for youth, the team, An Instant Gratification Situation, aims to develop content, stories, and messaging for social media platforms targeted toward youth for obtaining reproductive health services [51]. These platforms can be useful in engaging youth where they are—on mobile phones using social media—and continue to promote positive uses of social media platforms.

### Limitations

There are several limitations specific to this study. First, results are based on self-reported, personal data that could be subject to response bias due to social desirability resulting from participants completing surveys in their school/program environment. This was attenuated by research assistants, not affiliated with the program, administering the surveys. Additionally, the use of personal laptop computers and audio capability increases data dependability [4,28].

A benefit of longitudinal data is the ability to control for individual heterogeneity and measure effects that are not detectable in pure cross-sectional studies, but these types of data are also sensitive to loss to follow up [52,53]. Strategies to reduce attrition permitted an overall 4% response rate at the 16-month follow-up survey, which is an acceptable response rate [54].

Despite this study utilizing longitudinal data, it is still limited in that we did not measure or test every variable theorized to influence sexual risk behavior. The relationships in this study may also be based on confounding variables that were not measured, such as peer behaviors [55], older sexual partners [43], parent-youth discussions about sexual health behaviors [56], relationship status, or having experienced dating violence [57]. Finally, although social media crosses cultural boundaries, this study was limited to a Latino population in Maryland; so generalizability is cautioned. However, it remains an important study of an emerging topic surrounding adolescent’s social media use and how it affects their lives.

### Conclusions

Although adolescents exchange SMS at high rates, parental monitoring remains vital to parent-child relationships and can moderate the relationship between adolescents’ SMS frequency and sexual risk behaviors. When parents are involved in the lives of their adolescents, the extent of SMS sent or received does not influence risky sexual behaviors and suggests SMS is simply another form of communication that poses no additional threat.

## Abbreviations

CDC: Centers for Disease Control and Prevention
SES: socioeconomic status
SMS: short message service
SNS: social networking sites
STIs: sexually transmitted infections
